# Tobramycin exposure from active calcium sulfate bone graft substitute

**DOI:** 10.1186/2050-6511-15-12

**Published:** 2014-03-04

**Authors:** Françoise Livio, Peter Wahl, Chantal Csajka, Emanuel Gautier, Thierry Buclin

**Affiliations:** 1 Division of Clinical Pharmacology, Biomedicine, Department of Laboratories, Centre Hospitalier Universitaire Vaudois, Lausanne 1011, Switzerland; 2 Department of Orthopedic Surgery, Cantonal Hospital, Fribourg, Switzerland; 3 Department of Pharmaceutical Sciences, Clinical Pharmacy Unit, University of Geneva, Rue du Général- Dufour 24, Genève 4 1211, Switzerland

**Keywords:** Tobramycin, Bone graft substitute, Pharmacokinetics, Renal failure

## Abstract

**Background:**

Bone graft substitute such as calcium sulfate are frequently used as carrier material for local antimicrobial therapy in orthopedic surgery. This study aimed to assess the systemic absorption and disposition of tobramycin in patients treated with a tobramycin-laden bone graft substitute (Osteoset® T).

**Methods:**

Nine blood samples were taken from 12 patients over 10 days after Osteoset® T surgical implantation. Tobramycin concentration was measured by fluorescence polarization. Population pharmacokinetic analysis was performed using NONMEM to assess the average value and variability (CV) of pharmacokinetic parameters. Bioavailability (F) was assessed by equating clearance (CL) with creatinine clearance (Cockcroft CLCr). Based on the final model, simulations with various doses and renal function levels were performed. (ClinicalTrials.gov number, NCT01938417).

**Results:**

The patients were 52 +/− 20 years old, their mean body weight was 73 +/− 17 kg and their mean CLCr was 119 +/− 55 mL/min. Either 10 g or 20 g Osteoset® T with 4% tobramycin sulfate was implanted in various sites. Concentration profiles remained low and consistent with absorption rate-limited first-order release, while showing important variability. With CL equated to CLCr, mean absorption rate constant (ka) was 0.06 h-1, F was 63% or 32% (CV 74%) for 10 and 20 g Osteoset® T respectively, and volume of distribution (V) was 16.6 L (CV 89%). Simulations predicted sustained high, potentially toxic concentrations with 10 g, 30 g and 50 g Osteoset® T for CLCr values below 10, 20 and 30 mL/min, respectively.

**Conclusions:**

Osteoset® T does not raise toxicity concerns in subjects without significant renal failure. The risk/benefit ratio might turn unfavorable in case of severe renal failure, even after standard dose implantation.

## Background

Since the end of the sixties, bone cement such as polymethyl methacrylate (PMMA) and bone graft substitute such as calcium sulfate are frequently used as carrier material for local antimicrobial therapy in orthopedic surgery for osteomyelitis, infected arthroplasty, soft tissue infections or prophylaxis. Unlike PMMA, calcium sulfate is resorbable, thus obviating the need for surgical removal. Osteoset® T (Wright Medical Technology Inc, Arlington, TN, USA) is one of these products; it contains 4% tobramycin sulfate in calcium sulfate pellets. Most data on Osteoset® T come from animal studies, where high local and low systemic tobramycin concentrations have been observed [1]. No pharmacokinetic (PK) observations with patients treated with Osteoset® T have been published so far, despite well known concentration-related potential toxicity of aminoglycosides. Only concentrations have been described in patients implanted with a tobramycin-laden polymer carrier material (Simplex™ P Bone Cement with Tobramycin) showing high tobramycin levels at the operative site but low systemic absorption [2].

The aim of this study was to develop a population PK model for tobramycin in patients treated with an active calcium sulfate bone substitute and to predict tobramycin systemic exposure under various dose and renal function levels.

## Method

### Patients and sampling

The data were collected prospectively between October 2006 and March 2008 from all adult patients treated with Osteoset® T in our orthopedic surgery department. Osteoset® T was implanted whenever estimated a useful adjunct to standard therapy for bone, soft tissues and prosthetic joint infections requiring surgical debridement. Either 10 g or 20 g of Osteoset® T with 4% tobramycin sulfate were implanted, representing 262 or 524 mg tobramycin repectively taking into account the salt factor (tobramycin/tobramycin sulfate = 0.655). Additional intravenous aminoglycosides were not used in these patients. No wound drains were used.

Blood samples were taken at 3 h, 6 h, 12 h, 24 h, 48 h and on days 3, 5, 7 and 10 after implantation. Whenever a tourniquet had been used during the operation, its release-time was taken into account instead of implantation time. Blood was sampled in BD Vacutainer® SST II Advance (Becton Dickinson AG, Allschwil, Switzerland) serum separator tubes.

The study protocol was approved by the Institutional Review Board of Fribourg Cantonal Hospital and all subjects gave their written informed consent. This study was registered in ClinicalTrials.gov under code number NCT01938417.

### Drug assay

Tobramycin concentrations were measured on a COBAS INTEGRA 800 (Roche Diagnostics GmbH, Mannheim, Germany) fluorescence polarization detector, using a standard tobramycin measurement kit (ref. 20737925). The lower limit of quantification (LOQ) of the assay was 0.1 mg/L. Coefficient of variation (CV) within runs was 1.7% at 2.2 mg/L, while CV between runs were 6.2%, 2.6% and 2.5% at 0.9 mg/L, 2.8 mg/L and 4.5 mg/L, respectively. Accuracy was monthly checked by participating to the external quality control scheme provided by LGC Standards for antibiotics (LGC Standards Proficiency Testing, Bury, UK). Assays were processed within 6 hours after sampling.

### Population pharmacokinetic analysis

The pharmacokinetic analysis was performed using nonlinear mixed-effects modeling with the NONMEM computer program (version VI) [3]. Models were fitted to the data with the first-order conditional estimation with interaction (FOCE INTER) method. One and two compartment models with first-order absorption were compared. The first tobramycin concentration below the LOQ was set to half of the LOQ value and subsequent points below the LOQ were dropped out. Average value and variability of clearance (CL), volume of distribution (V) and absorption rate constant (k_a_) were assessed. As aminoglycosides are known to be completely and exclusively eliminated by glomerular filtration, tobramycin CL was equated to the creatinine clearance (CL_cr_) value, thus enabling the estimation of absolute bioavailability (F) in the absence of intravenous injection. CL_cr_ was estimated with the Cockcroft formula, based on patient’s serum creatinine, body weight, and sex [4], with linear interpolation for days without creatinine measurement. The residual variability was adequately modeled using an additive plus proportional error model. The final model was then developed by testing in a stepwise fashion the potential influence of sex, age, body weight, Osteoset® T quantity and implantation site on the pharmacokinetic parameters.

Based on the final average parameters and variability, simulations using various tobramycin doses (262 mg; 786 mg; 1310 mg) at various CL_cr_ levels (120, 90, 60, 30, 20 and 10 mL/min) were performed, using 2000 virtual patients each to predict the doses associated with concentrations exceeding the 2 mg/L threshold over a prolonged period of time (5 days or more).

## Results

Twelve patients (7 males, 5 females) were included in this observational study. Their mean age was 52 years (range 19–82, SD ± 20), body weight 73 kg (53–116, ± 17), and CL_cr_ 119 mL/min (34–288, ± 55).

Osteoset® T implantation sites were tibia/fibula (6 patients), hip (2), calcaneum (2), femur (1) and lumbar spine (1), either for established infections (9) or prophylaxis (3). Eight patients were implanted 10 g Osteoset® T containing 262 mg tobramycin, while 4 were implanted 20 g Osteoset® T containing 524 mg tobramycin.

**Figure 1:**
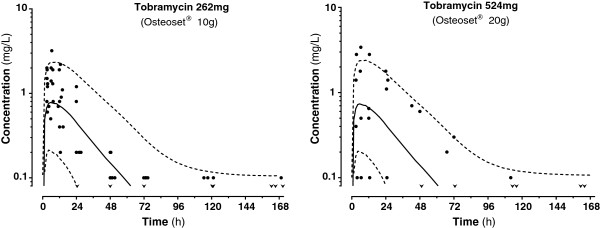
**Concentrations versus time plot of tobramycin with mean population prediction (solid line) and 90% prediction interval (dashed lines) after Osteoset® 10 g (left) or 20 g (right) in patients with normal renal function.** Dose difference is almost fully compensated by different bioavailability, giving similar predictions. Superimposed points show the concentrations observed in 8 patients after 10 g and 4 patients after 20 g. v : first concentration below the limit of detection.

Osteoset® T was well tolerated; further clinical details have been presented elsewhere [5]. Systemic tobramycin concentrations measured remained low (<2 mg/L at 24 h) in all patients. Concentration profiles were consistent with a one compartement model with absorption rate-limited first-order release, while showing important variability. A two compartment model was also tested but did not improve the fit. With CL equated to CL_Cr_, mean estimated k_a_ was 0.0603 h^−1^, volume of distribution 16.6 L (CV 89%) and F was 63% and 32% (CV 74%) in the 8 patients having received 10 g Osteoset® T and in the 4 patients having received 20 g, respectively. Sex, age, body weight or implantation site did not seem to affect tobramycin absorption or disposition when introduced as covariates. Population pharmacokinetic parameter estimates and variability are summarized in Table 1. The concentration values observed are represented on Figure 1, along with mean population prediction and 90% prediction intervals for CL_Cr_ 120 mL/min.

**Table 1:** Tobramycin population pharmacokinetic parameter estimates

**Population mean**		
**Parameter**	**Estimate**	**s.e%**^***b***^
CL (L h^−1^)	7.14^*d*^	--
V (L)	16.6	35
K_a_ (h^-1)^	0.0603	19
F (if cast 10 g)	0.63	19
F (if cast 20 g)	0.32	19
**Interpatient variability**		
**Parameter**	**CV%**^***a***^	**s.e%**^***c***^
CL	--	--
V	89	74
k_a_	--	--
F	74	72
**Residual variability**		
**Error type**		**s.e%**
σ_prop_ (CV%)^*e*^	29	50^*c*^
σ_add_ (SD in mg/L)^*f*^	0.062	22^*b*^

CL, mean apparent clearance; V, mean apparent volume of distribution; k_a_, mean absorption rate constant; F, mean bioavailability.

^*a*^estimates of variability expressed as coefficient of variation (CV%).

^*b*^s.e = standard error of the estimates (S.E), defined as S.E/estimate and expressed as percentage.

^*c*^s.e = standard error of the coefficient of variations, taken as $\sqrt{s.e_{\mathrm{estimate}}/\mathrm{estimate}}$, expressed as a percentage.

^*d*^equated to creatinine clearance (CL_cr_).

^*e*^residual variability in the plasma concentrations associated with the proportional error term, expressed as a coefficient of variation (CV%).

^*f*^residual variability in the plasma concentrations associated with the additive error term, expressed as a standard deviation (SD).

The simulations of concentration profiles (using an average F estimate of 47%) showed that more than 5% patients would maintain concentrations over 2 mg/L during at least five days post implantation with tobramycin 262, 786 and 1310 mg when CL_cr_ was respectively set at 10, 20 and 30 mL/min.

## Discussion and conclusions

This is the first tobramycin pharmacokinetic study after Osteoset® T implantation in a clinical setting. Tobramycin systemic concentration values were measured well below the traditional toxicity threshold of 2 mg/L from 24 h and later on, which was not unexpected considering the low tobramycin dose implanted and the good renal function of the study subjects. Whether continuous systemic exposure to low concentrations below minimal inhibitory concentration may favor antimicrobial resistance remains an issue [6].

Prominent inter-individual variability was observed, probably due to heterogeneity of patients, indications, surgical techniques and implantation sites. There have probably been too few patients included for reliably testing the influence of all covariates. Loss through wound oozing has been neglected (not measured) but could represent another source of variability. Imprecision on low tobramycin levels measurement, very close to the LOQ, could also have increased residual variability.

The significant difference in bioavailability between 10 g and 20 g Osteoset® T (63% versus 32% respectively) could indicate that higher amounts of bone graft substitute either limit tobramycin release to some extent, or slow it down sufficiently for the resulting concentrations to fall below the LOQ. Unbalanced loss through wound oozing could also partly account for this difference. Implantation site and tissue perfusion could also play a role, although this could not be demonstrated in our analysis, due to limited data.

Our model gained in credibility when tobramycin CL was equated to CL_cr_; indeed there is no argument to think of a different elimination route for the drug absorbed from Osteoset® T compared to intravenous delivery [7]. The Cockcroft equation that we used has yet its limitations, in particular in obese or bedridden patients. Our model found a distribution volume of 0.22 L/kg body weight, consistent with previously published values [7]. The large inter-patient variability found in both V and F is likely to incorporate some amount of variability in CL and k_a_.

High sustained potentially toxic concentrations were predicted by simulations with standard doses Osteoset® T at renal failure stages 4 and 5. Considering the limited study power, this simulation should be taken as a rough estimate of the potential for toxicity of this product. Our model is probably too imprecise to deduce precise dose adjustment guidelines. However, it indicates that caution is warranted when Osteoset® T implant is considered for patients with severe renal failure, as its benefit/risk ratio could turn out unfavourable [8].

## Competing interests

The authors declare that they have no competing interests.

## Authors’ contributions

FL performed the pharmacokinetic analysis and was involved in drafting the manuscript. PW designed the study and was involved in the acquisition of data. CC revised the pharmacokinetic analysis. EG was involved in the study design and coordination. TB gave final approval of the version to be published. All authors read and approved the final manuscript.

## Pre-publication history

The pre-publication history for this paper can be accessed here:

http://www.biomedcentral.com/2050-6511/15/12/prepub
